# Cdc2-like kinase 2 is a key regulator of the cell cycle via FOXO3a/p27 in glioblastoma

**DOI:** 10.18632/oncotarget.8471

**Published:** 2016-03-30

**Authors:** Soon Young Park, Yuji Piao, Craig Thomas, Gregory N. Fuller, John F. de Groot

**Affiliations:** ^1^ Department of Neuro-Oncology, The University of Texas MD Anderson Cancer Center, Houston, Texas, USA; ^2^ Department of Pathology, The University of Texas MD Anderson Cancer Center, Houston, Texas, USA

**Keywords:** CLK2, glioma, cell cycle, FOXO3a, p27

## Abstract

Cdc2-like kinase 2 (CLK2) is known as a regulator of RNA splicing that ultimately controls multiple physiological processes. However, the function of CLK2 in glioblastoma progression has not been described. Reverse-phase protein array (RPPA) was performed to identify proteins differentially expressed in CLK2 knockdown cells compared to controls. The RPPA results indicated that CLK2 knockdown influenced the expression of survival-, proliferation-, and cell cycle-related proteins in GSCs. Thus, knockdown of CLK2 expression arrested the cell cycle at the G1 and S checkpoints in multiple GSC lines. Depletion of CLK2 regulated the dephosphorylation of AKT and decreased phosphorylation of Forkhead box O3a (FOXO3a), which not only translocated to the nucleus but also increased p27 expression. In two glioblastoma xenograft models, the survival duration of mice with CLK2-knockdown GSCs was significantly longer than mice with control tumors. Additionally, tumor volumes were significantly smaller in CLK2-knockdown mice than in controls. Knockdown of CLK2 expression reduced the phosphorylation of FOXO3a and decreased Ki-67 *in vivo*. Finally, high expression of CLK2 protien was significantly associated with worse patient survival. These findings suggest that CLK2 plays a critical role in controlling the cell cycle and survival of glioblastoma via FOXO3a/p27.

## INTRODUCTION

Glioblastoma is a highly malignant, aggressive disease that is the most common primary intrinsic brain tumor in adults. CSNK2A1, the gene that encodes for CK2a, is amplified in glioblastoma [1]. CK2 can positively regulate phosphoinositide 3-kinase (PI3K)/AKT activity by directly phosphorylating AKT at Ser129 [2]. Kim et al. [3] found that CX-4945 is a potent, selective inhibitor of CK2 that modulates serine/arginine-rich protein phosphorylation by directly inhibiting Cdc2-like kinases (CLKs). As splicing factors, the serine/arginine-rich proteins play essential roles in alternative as well as constitutive splicing [4]. The CLKs, including CLK1, CLK2, and CLK3 [5-7], phosphorylate serine/arginine-rich proteins, which regulate RNA splicing [3]. Inhibitors of CLKs suppress cell growth and induce apoptosis by modulating pre-mRNA splicing [8]. In particular, CLK2 is an insulin-regulated suppressor of hepatic gluconeogenesis [9]. In the refed state, hepatic CLK2 protein level and kinase activity is induced by insulin/AKT signaling. Phosphorylation of CLK2 at Ser34 and Thr127 by AKT controls cell survival after ionizing radiation. Furthermore, AKT directly binds to CLK2 and phosphorylates CLK2 [10].

Many kinase pathways activate Forkhead box O (FOXO) transcription factors, such as stress-activated c-Jun NH2 kinase [11, 12], AMP-activated protein kinase [13, 14], and PI3K/AKT. Phosphorylated AKT regulates phosphorylation of FOXO3a, including at Thr32, Ser253, and Ser315, which increases translocation of FOXO3a from the nucleus to cytosol and deregulation of FOXO3a function [15, 16]. IκB kinase β and extracellular signal-regulated kinase phosphorylate FOXO3a, as well. The translocation of FOXO3a to cytosol leads to ubiquitination and proteasomal degradation. FOXO expression has been downregulated in cases of several types of cancer, such as breast cancer, prostate cancer, glioblastoma, and leukemia [17, 18]. Four FOXO factors (FOXO1, FOXO3, FOXO4, and FOXO6) regulate apoptosis [19, 20], the cell cycle [21, 22], and DNA damage repair [23]. FOXO3a translocated to the nucleus functions with increased levels of p27^kip1^ and Bim expression, allowing for regulation of the cell cycle and cell death, respectively. In addition, cyclin-dependent kinases (CDKs) are heterodimeric serine/threonine kinases that control cell division [24]. CDK complexes are inhibited by CDK inhibitors such as INK4 and kinase-inhibiting proteins, the latter of which consist of p21^cip1^, p27^kip1^, and p57^kip2^. In particular, p27^kip1^ can negatively regulate cyclin E/CDK2 and block progression of the cell cycle from G1 to S phase [25].

In previous studies, understanding of CLK2 targets and function except as a splicing factor was limited. In the present study, we examined newly discovered function of CLK2 in glioblastoma stem cells (GSCs) *in vitro* and *in vivo*. Knockdown of CLK2 expression in several GSC lines correlated with AKT/FOXO3a/p27 and arrest of the cell cycle at G1 and S phase *in vitro* and extended survival durations in orthotopic xenograft models of glioblastoma.

## RESULTS

### Expression of CLK2 regulates the proliferation and viability of GSCs

**Figure 1:**
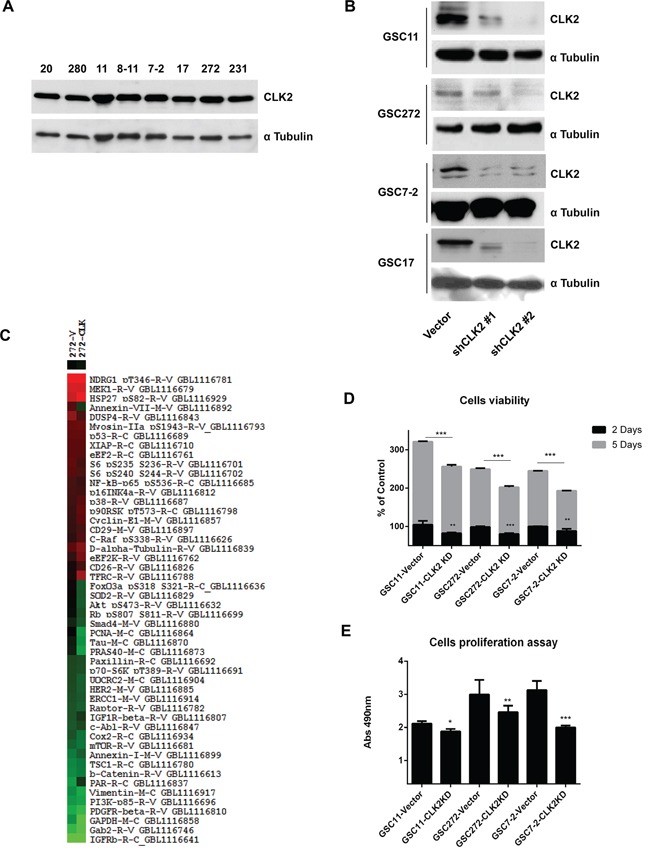
Role of CLK2 expression in GSCs **A,** GSC lysates were collected, and CLK2 expression in the lysates was detected using Western blot analysis. **B,** GSC11, GSC272, GSC7-2 and GSC17 cells were infected with a lentivirus containing a control vector, CLK2 shRNA1, or CLK2 shRNA2. **C,** Hierarchical clustering was applied to protein expression data from RPPA results in vector shRNA and CLK2 shRNA in GSC272 cells. The protein expression levels in CLK2 shRNA-infected cells differed at least 1.6-fold from those in control GSC272 were selected for hierarchical clustering analysis. The red and green colors in the cells reflect relatively high and low expression, respectively. **D,** the cells were infected with a lentivirus containing a control vector or CLK2 shRNA, a volume of CellTiter-Glo Reagent was added to cells, and the luminescence was recorded. **E,** the GSCs per well were added to a 96-well plate with 20 μl of a combined MTS/PMS solution, and the plate was incubated for 3h 30min at 37°C in a humidified atmosphere. *P < 0.05; **P < 0.01; ***P < 0.001 versus control.

To investigate the regulation of CLK2 expression in GSCs, we first performed Western blot analysis of several GSC lines and observed expression of CLK2 in all of them (Figure 1A). Stable knockdown of CLK2 expression in GSCs by infection with specific shRNA-containing lentiviruses abrogated CLK2 expression in the cells infected with shRNA2 construct compared to those infected with the shRNA1 construct (Figure 1B, Supplementary Figure S1). We first analyzed CLK2 function in CLK2-knockdown and vector-infected GSC272 cells using RPPA (Figure 1C and Supplementary Figure S2). The RPPA data demonstrated that CLK2 expression correlated with proteins involved in cell proliferation, growth, and survival. We confirmed the RPPA results that knockdown of CLK2 reduced cell viability and proliferation in several GSCs (Figure 1D and 1E). At 2 and 5 days after seeding, knockdown of CLK2 expression decreased the viability of GSC11, GSC272, and GSC7-2 cells (Figure 1D). Also, knockdown of CLK2 expression decreased the proliferation of GSC11, GSC272, and GSC7-2 cells (Figure 1E). CX-4945, inhibitor of CK2, also decreased cell viability in GSC11, GSC272, GSC7-2 cells (Supplementary Figure S3).

### Knockdown of CLK2 expression is associated with cell-cycle arrest at G1 and S phase and upregulation of p27 expression

**Figure 2:**
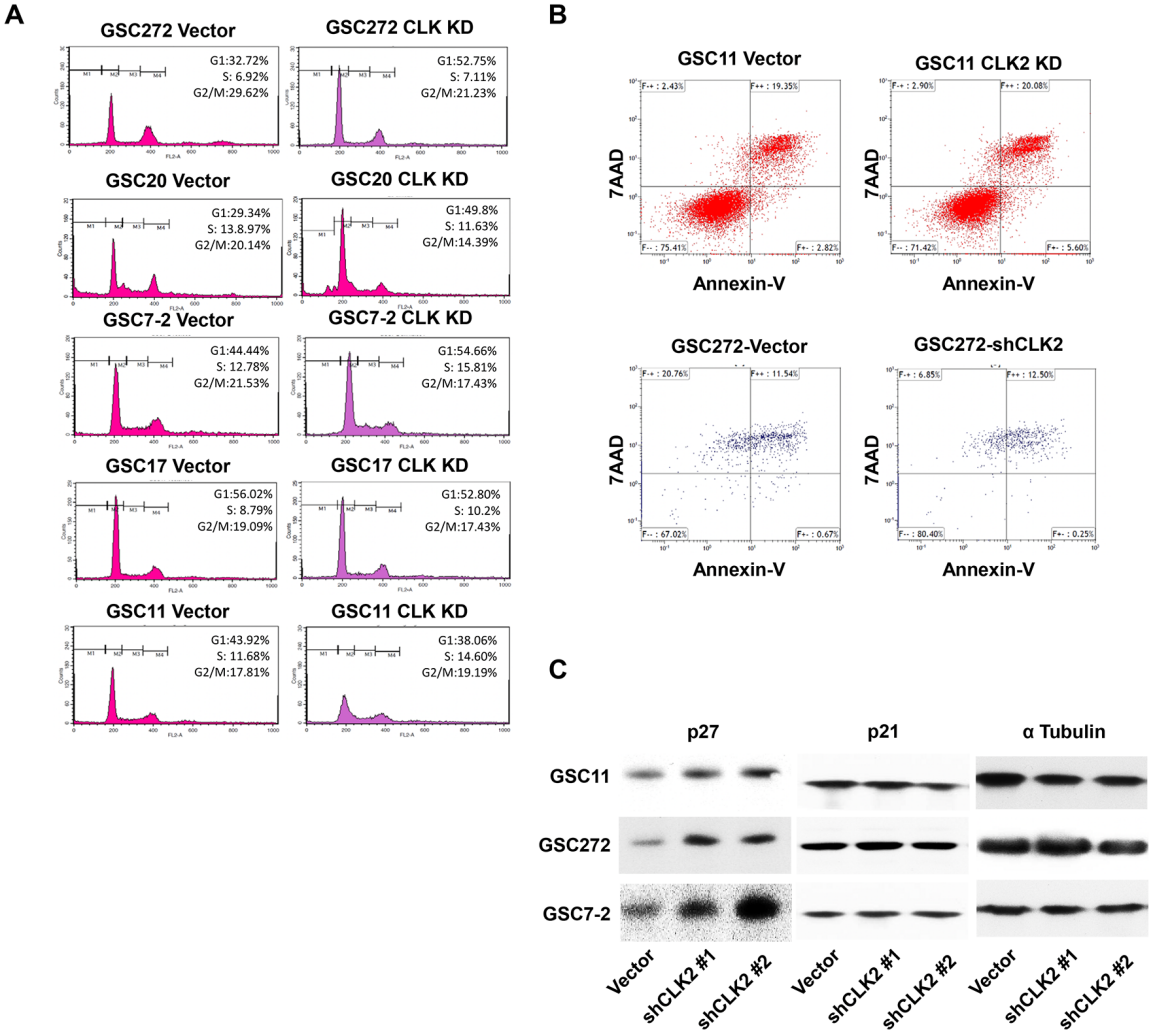
Knockdown of CLK2 expression arrests the cell cycle at G1 and S phase and increases p27 expression in GSCs **A,** GSCs were incubated with 70% EtOH at -20°C for 1 day and washed three times with PBS and a propidium iodide staining solution. **B,** GSCs were incubated with bFGF and EGF in B27 media for 3 days, and single cells were treated with Accutase. The cells were stained with annexin V and 7-AAD, and apoptosis of the cells was detected using FACS analysis. **C,** after knockdown of CLK2 expression, expression of p27 protein in several GSC lines was measured using Western blotting.

Cell-cycle progression is tightly regulated by cell cycle-regulatory proteins such as CDKs and CDK inhibitors [26, 27]. Our RPPA results suggested that CLK2 may regulate cell-cycle progression, as well. We performed flow cytometry using propidium iodide staining to determine the effect of CLK2 on the cell-cycle in GSCs. We found that after knockdown of CLK2 expression, the cell cycle was arrested in G1 with the number of cells in G1 increasing from 32.72 to 52.75% in GSC272, from 21.34 to 49.8% in GSC20, and from 44.44 to 54.66% in GSC7-2 (Figure 2A). Also, the cell cycle was arrested in S phase where the number of cells increased from 8.79 to 10.20% in GSC17 cells and from 11.68 to 14.60% in GSC11 cells (Figure 2A). In contrast, CLK2 expression had no effect on apoptosis in GSC11 or GSC 272 cells (Figure 2B). p21 and p27 are key regulator of the cell cycle from G1 to S phase [25]. p27 protein expression was elevated in several GSCs after CLK2 knockdown (Figure 2C). However, CLK2 did not change the expression of p21.

### CLK2 regulates the phosphorylation of AKT/FOXO3a in GSCs

**Figure 3:**
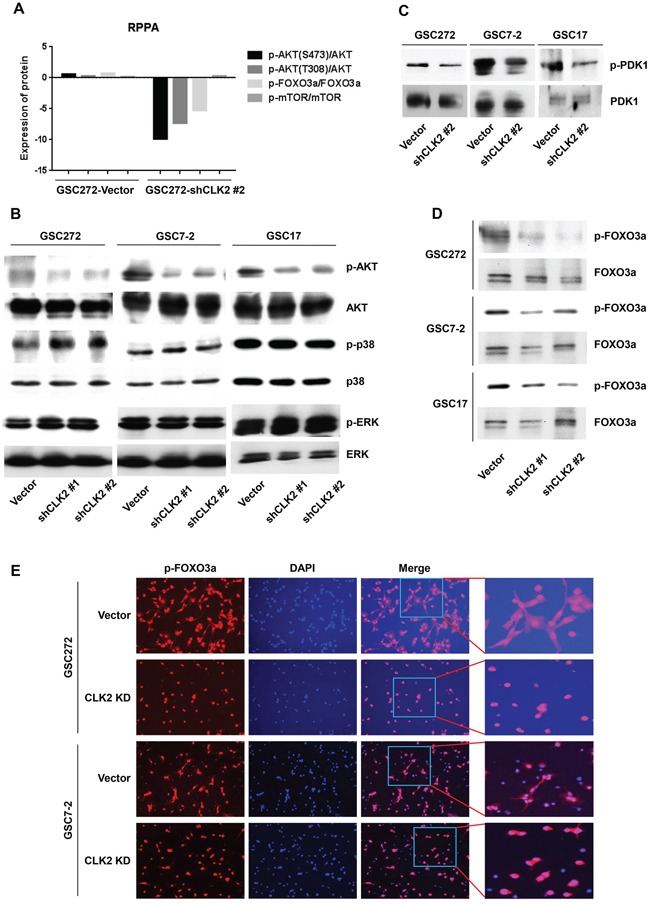
CLK2 regulates the phosphorylation of AKT and FOXO3a protein **A,** the graph shows that the phosphorylation for AKT, FOXO3a, and mTOR normalized total expression of AKT, FOXO3a, mTOR in vector- and CLK2 shRNA-infected GSC272 cells. The gene expression levels were determined using RPPA analysis. **B** and **C,** GSCs of vector and CLK2 knockdown were incubated with bFGF and EGF for 1 hour. The cells were harvested, lysed, and analyzed using Western blotting. **D** and **E,** GSCs were incubated with bFGF and EGF for 24 hours. Phosphorylation of FOXO3a was assessed using Western blotting **D,** and immunofluorescence **E.** Phosphorylated FOXO3a stained red, and the nuclei were counterstained with 4′,6-diamidino-2-phenylindole (blue).

CLK2 is a negative regulator of the activity of the signaling protein AKT [28]. According to our RPPA results, expression of phosphorylated AKT (Ser473/Thr308) and FOXO3a were lower in CLK2-knockdown than in vector-infected GSC272 cells (Figure 3A). More interestingly, GSC272 cells with depletion of CLK2 exhibited no changes in mTOR expression. Depletion of CLK2 regulated dephosphorylation of AKT in several GSCs, whereas phosphorylation of extracellular signal-regulated kinase and p38 mitogen-activated protein kinase was unchanged in GSC7-2, and GSC17 cells and slightly increased in GSC272 cells (Figure 3B). We also examined PDK1 upstream of AKT and found that CLK2 regulated the phosphorylation of PDK1 (Figure 3C). Furthermore, FOXO3a expression is suppressed by AKT phosphorylation and cytoplasmic sequestration [16], and FOXO3a signaling pathways may be downstream regulation of CLK2. These data are consistent with our results, as the level of phosphorylation of FOXO3a was lower in CLK2-knockdown GSC7-2, GSC17, and GSC272 cells than in control cells (Figure 3D). In the control GSC7-2 and GSC272 cells, phosphorylated FOXO3a was expressed in both the cytoplasm and nucleus, whereas in the CLK2-knockdown cells, it was expressed only in the nucleus (Figure 3E). We also sought to determine the involvement of FOXO3a via AKT signaling in GSCs. Using inhibitors of AKT (AKTi) and mTOR (rapamycin), we observed reduced FOXO3a phosphorylation and increased p27 expression in GSC7-2 and GSC272 (Supplementary Figure S4). Collectively, these observations demonstrated that CLK2 deficiency leads to regulation of AKT/FOXO3a/p27 signaling and contributes to cell-cycle arrest at G1 and S phase.

### CLK2 knockdown reduces glioblastoma tumor growth *in vivo* and prolongs survival

**Figure 4:**
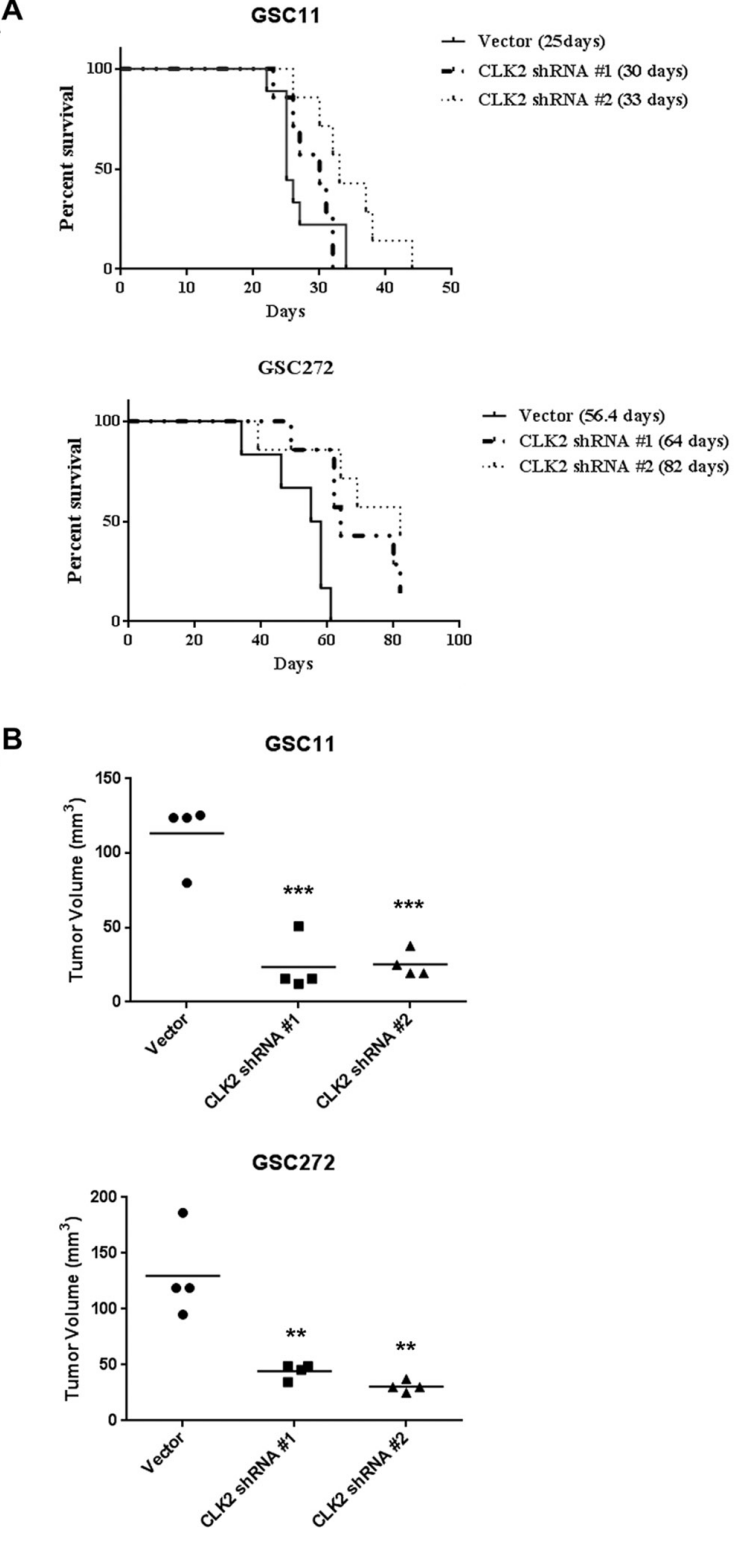
Knockdown of CLK2 expression prolongs survival and decreases tumor volume in glioblastoma mouse models The effects of targeting CLK2 were examined using an intracranial xenograft model of glioblastoma. GSC272 and GSC11 cells were infected with a lentivirus containing an empty vector, CLK2 shRNA1, or CLK2 shRNA2 and then implanted in the brains of immunocompromised mice (5 × 10^5^ cells/mouse). **A,** Kaplan-Meier curves of the estimated survival durations in the study mice. In the GSC11 group, the following were compared: control and CLK2 shRNA-infected cell implantation (shRNA1, *P =* not significant; shRNA2, *P* = 0.0278). In the GSC272 group, the following were compared: control versus CLK2 shRNA-infected cell implantation (shRNA1, *P* = 0.035; shRNA2, *P* = 0.046). **B,** hematoxylin and eosin stains of brains harvested from the study mice at 32 (GSC11) or 60 (GSC272) days after GSC implantation. Mice of implanted cells with knockdown of CLK2 expression produced smaller tumors than did control cells (**P < 0.01; ***P < 0.001 versus control).

We performed orthotopic mouse xenograft experiments to determine the effect of CLK2 expression on glioblastoma progression *in vivo*. We injected CLK2-knockdown GSC11 and GSC272 cells into the brains of nude mice. We found that the median survival durations were 82.0, 64.0, and 56.4 days in mice injected with shRNA2-infected cells, shRNA1-infected cells, and vector cells, respectively (Figure 4A). In the GSC11-injected mice, only injection of shRNA2-infected cells produced markedly longer survival durations than did injection of vector cells. As in our *in vitro* assay, construct of shRNA2 infected cells had a greater effect on survival curve than did shRNA1-infected cells *in vivo* study. Furthermore, the volumes of CLK2-deficient shRNA1- and shRNA2-infected tumors were much lower than those of control tumors in GSC11 and GSC272 mouse models (Figure 4B).

### Knockdown of CLK2 expression suppresses Ki-67 and phosphorylated AKT/FOXO3a expression

**Figure 5:**
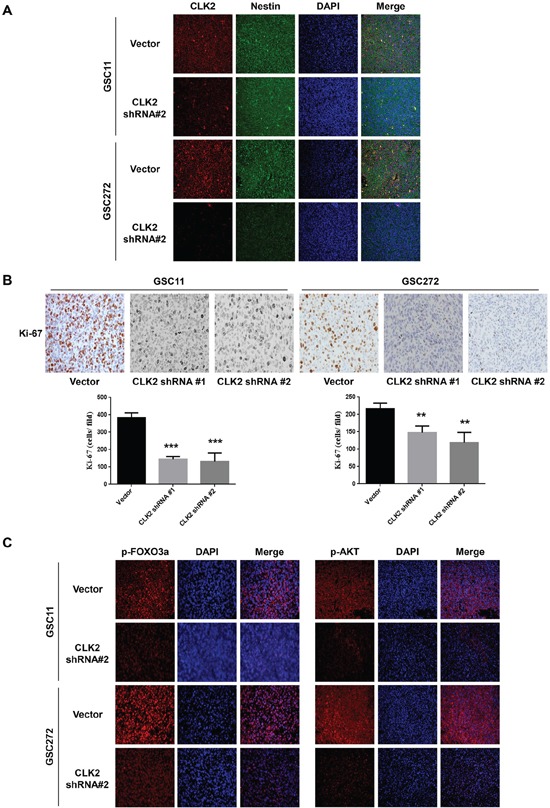
Depletion of CLK2 decreases the expression of Ki-67 and phosphorylation of AKT/FOXO3a **A-B.** immunohistochemical stains of GSC11 and GSC272 tumor samples obtained from orthotopic murine xenografts. A, immunofluorescent stains for CLK2 and the stem cell marker nestin in GSC11 and GSC272 mouse xenografts with or without infection with CLK2 shRNA are shown. CLK2 was labeled as red and nestin as green. Expression of CLK2 and nestin overlap. **B,** Ki-67–positive cells stained brown with hematoxylin counterstain of nuclei. The bar graph shows the numbers of Ki-67–positive cells in vector-infected and CLK2-knockdown xenografts. **C,** immunofluorescent stains for phosphorylated AKT and FOXO3a in murine GSC11 and GSC272 cells. Phosphorylated AKT and FOXO3a stained red, and the nuclei were counterstained with 4′,6-diamidino-2-phenylindole (blue).

Because of the CLK2-mediated effect on glioblastoma, we examined the expression of several proteins in the tumor xenografts from GSC272 and GSC11 cells. To determine the location of the expression of CLK2, we performed double-staining for the stem cell marker nestin and CLK2. We observed that CLK2 colocalized with nestin (Figure 5A). Immunohistochemical analysis of tumor sections obtained from CLK2-knockdown tumors for Ki-67 demonstrated decreased expression of cells proliferating, whereas sections obtained from vector-infected mice had high expression of Ki-67 (Figure 5B). Immunofluorescent analysis of slides obtained from vector-infected mice demonstrated higher expression of phosphorylated AKT and FOXO3a than that on tissue sections from CLK2-knockdown mice in the GSC11 and GSC272 models (Figure 5C). These data indicated that CLK2 deficiency correlated with reduced *in vivo* tumor growth and prolonged survival, which are regulated by phosphorylation of AKT/FOXO3a and Ki-67 expression as a marker of proliferation. We also found that CLK2 is amplified and overexpressed in human glioblastomas and that its downregulation inhibits glioblastoma cell growth in cell culture models and glioblastoma tumorigenesis in a xenograft assay.

### Increased expression of CLK2 in gliblatoma tissue correlates with poor outcome

**Figure 6:**
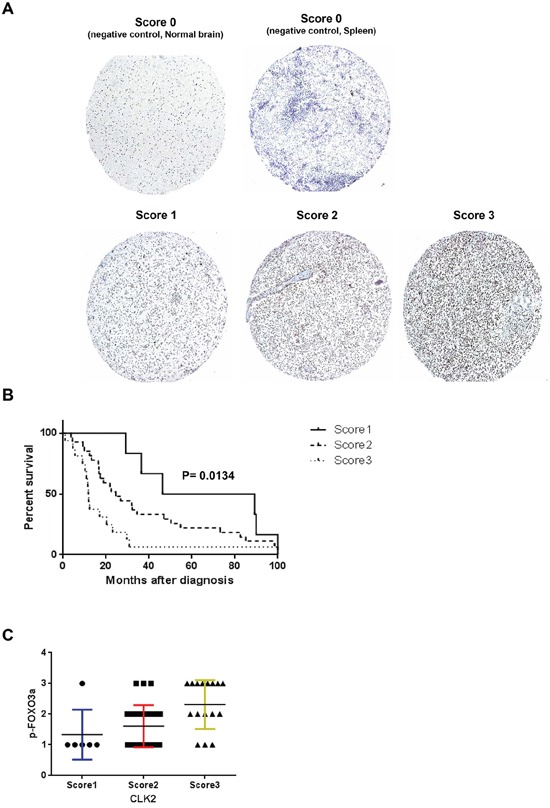
Expression of CLK2 in human glioblastoma specimens is inversely correlated with patient outcome **A,** immunohistochemical analysis of CLK2 expression in human glioblastoma specimens. CLK2 expression was independently validated using a glioblastoma tissue microarray. Representative immunohistochemical images of overall CLK2 expression in glioblastoma tissue microarray are shown (magnification, x100). Tissue punches were scored for CLK2 expression as 0 (negative/absent expression), 1 (weak expression), 2 (moderate expression), or 3 (strong expression). **B,** Kaplan-Meier survival analysis according to CLK2 expression by immunohistochemistry and log rank test was used to test for significant differences. **C,** immunohistochemical analysis of FOXO3a phosphorylation in the glioblastoma tissue microarray. Phosphorylation of FOXO3a was positively correlated with CLK2 expression.

We performed immunohistochemical analysis of 50 glioblastoma, 2 normal spleen and normal brain tissues in a tissue microarray. We observed moderate (grade 2) and strong (grade 3) staining for CLK2 in 28 (56%) and 16 (32%) glioblastoma samples, respectively (Figure 6A). None of the glioblastoma tissue samples had a zero (grade 1) intensity score. The staining pattern was primarily nuclear on the tissues in the microarray. Log-rank test analysis revealed a significant (p=0.0134) inverse association between CLK2 intensity score and increased survival duration (Figure 6B). In order to clarify the correlation of CLK2 expression and phosphorylation of FOXO3a, we stanined the TMA for phosphorylated FOXO3a. When sorted according to CLK2 staining intensity score, FOXO3a phosphorylation showed similar treads (Figure 6C).

## DISCUSSION

The CLK family kinases are dual specific kinases as phosphorylation of protein substrates on serine, threonine and tyrosine residues. Regulation of mRNA splicing is now recognized as a dynamic processing event, and CLK kinases identified as splicing factors *in vivo* [29, 30]. We report herein the identification of a function of CLK2 in glioblastoma stem cells. Specifically, knockdown of CLK2 expression correlated with proteins involved in glioms stem cell survival, proliferation, and the cell-cycle. Our results revealed that knockdown of CLK2 decreased GSCs viability *in vitro* and was accompanied by prolonged survival *in vivo*. p53 is a transcription factor that is induced by DNA damage, hypoxia and oncogene activator and induce cell cycle arrest and apoptosis [31]. Although knockdown of CLK2 did not change p53 expression, it did suppress the cell cycle at the G1 phase and decreased cell proliferation. In the xenograft models, expression of the proliferation maker Ki-67 decreased in CLK2-knockdown mice implanted with GSC11 and GSC272 tumor cells.

In the PI3K pathway, AKT is rapidly phosphorylated on activation loop residue T308 by PDK1 and in the hydrophobic region on S473. On AKT, PP2A and PP1 phosphatases can dephosphorylate the T308 and S473 sites [32]. CLK2 triggers AKT dephosphorylation via the PP2A phosphatase complex [28]. AKT binds directly to CLK2 and phosphorylates it *in vitro*, suggesting that CLK2 is an AKT substrate [10]. In our study, knockdown of CLK2 expression decreased phosphorylation of AKT and PDK1. In response to insulin signaling via PI3K/AKT, AKT phosphorylates the activation loop of CLK2 protein. Phosphorylation of the activation loop induces CLK2 kinase activity, which is required for CLK2 stability [9]. CLK2 can function as a feedback loop involved in AKT signaling. Studies elucidating a potential feedback loop between AKT and CLK2 in glioblastoma are needed.

The PI3K pathway is upregulated in glioma and involves amplification and/or overexpression of growth factors. In addition, it has been reported that reported that 88% of glioblastomas had altered PI3K/AKT signaling [33]. Other studies demonstrated that activated AKT induced FOXO3a accumulation in the cytoplasm from nucleus, whereas inhibition of AKT resulted in FOXO3a accumulation in the nucleus [16, 34]. Nuclear FOXO3a can arrest the cell cycle via p27 activation. We identified that inhibition of CLK2 expression using shRNA decreased phosphorylated FOXO3a *in vitro* and *in vivo*. We also observed that inhibition of AKT decreased phosphorylation of FOXO3a. In vector-infected GSCs, phosphorylated FOXO3a localized in the nucleus and cytosol, whereas in CLK2-knockdown cells, phosphorylated FOXO3a localized in the nucleus only.

FOXO3a induces differentiation and inhibits the self-renewal and tumorigenicity of glioblastoma cancer stem-like cells [35]. Also, FOXO3a deficiency impairs the leukemia-initiating potential of leukemia-initiating cells [36]. In our study, CLK2 deficiency markedly decreased glioblastoma tumor volume at 4.5 weeks (GSC11) and 8.5 weeks (GSC272) after implantation in mice. FOXO expression is dysregulated in several types of breast [37] and prostate [38] tumors. It has been previously been demonstrated that FOXO3a expression in human glioma samples is correlated with tumor grade. Researchers also found that FOXO3a staining was a similarly significant predictor of survival in grade II and III glioma cases [39]. For the first time, we CLK2 expression levels correlate with patient survival. Highly expressed CLK2 was associated with poor outcome in glioblastoma. Interestingly, a similar pattern of FOXO3a phosphorylation was observed as CLK2 expression.

The cell cycle is regulated by CDKs via a specific cyclin protein [40]. The kinase activity of CDK and cyclin complexes is required regulation of the cell cycle. Cyclin-dependent kinase inhibitoris have an important mechanism of regulation of cyclin/CDK activity. During the cell cycle, the CDK inhibitors p21 and p27 bind to cyclin/CDK complexes to inhibit their catalytic activity and induce cell-cycle arrest [41, 42]. A major activator of G1/S transition is AKT, which plays a role in the regulation of G1/S transition inhibitors such as p21 and p27 [43, 44]. In our study, the levels of p27 expression were markedly higher in CLK2-knockdown GSCs than in vector-infected cells. In contrast, the levels of p21 expression did not differ. Furthermore, inhibition of the AKT and mTOR signaling increased p27 expression. CLK2 regulated p27 expression via AKT phosphorylation.

In summary, we found that knockdown of CLK2 expression regulates cell-cycle arrest at G1 and S phase via dephosphorylation of AKT, which leads to dephosphorylation of FOXO3a and its downstream targets, such as p27. CLK2 is expressed in glioblastoma cells and human glioblatoma specimens, and knockdown of CLK2 expression not only enhances the survival of mice with glioblastoma but also decreases tumor growth. This study is the first to provide evidence of the function of CLK2 in the cell cycle in glioblastoma.

## MATERIALS AND METHODS

### Cell lines and reagents

GSC lines were isolated from brain tumor specimens at The University of Texas MD Anderson Cancer Center, Houston, TX. Acquisition of these cell lines was approved by the Institutional Review Board of MD Anderson Cancer Center. Glioma stem cell development is funded by the MD Anderson Brain Cancer SPORE supported by P50CA127001. GSCs were cultured in Dulbecco's modified Eagle's medium-F12 medium (1:1) with B27 (Invitrogen), basic fibroblast growth factor (bFGF; Sigma), and epidermal growth factor (EGF; Sigma) at 37°C in a humidified atmosphere of 5% CO_2_ and 95% air. GSCs were tested and authenticated by DNA typing at the MD Anderson Cancer Center Cell Line Characterization Core and were subsequently verified for our study (December 2014). 293T embryonic kidney cells were obtained from the American Type Culture Collection (ATCC) and maintained in Dulbecco's modified Eagle medium (DMEM) (Sigma) supplemented with 10% fetal bovine serum (FBS). All cell lines were free of mycoplasma contamination. Inhibitors of AKT (AKTi) were purchased from Calbiochem and mammalian target of rapamycin (mTOR) (rapamycin; cat. #S1039), casein kinase 2 (CK2) (CX4945; cat S2248) were purchased from Selleckchem.

### Stable knockdown of CLK2 expression in GSCs

293T cells were transfected with short hairpin RNA (shRNA), pMD2G, and pCMVR8.74 DNA using PolyJet reagents (cat. #SL100688; SignaGen) according to the manufacturer's instructions. An empty control vector (pGIPZ; cat. #RHS4349), CLK2 shRNA1 (cat. #V3LHS_635378-5), and CLK2 shRNA2 (cat. #V3LHS_365008-3) were purchased from GE Healthcare. GSCs were infected with a virus soup and selected for infected cells with puromycin (1 μg/ml) for 7 days. Cells were seeded one cell per well in a 96-well plate, and high-efficiency cells for further analysis.

### Cell viability and proliferation assay

For a cell viability assay, 5 × 10^3^ vector-infected and CLK2-knockdown GSCs per well were cultured for 2 and 5 days in a 96-well plate. Cell viability analysis was performed using a CellTiter-Glo Luminescent Cell Viability Assay (Promega) according to the manufacturer's protocol. For a cell proliferation assay, 1 × 10^5^ vector-infected and CLK2-knockdown cells per well added 20 μl of a combined MTS/PMS solution from a CellTiter 96 Aqueous Non-Radioactive Cell Proliferation Assay kit (Promega) in a 96-well plate, and the plate was incubated for 3h 30min at 37°C in a humidified atmosphere. The absorbance of cells was recorded at 490 nm using an enzyme-linked immunosorbent assay plate reader.

### Cell-cycle analysis and apoptosis assay

Vector-infected and CLK2-knockdown GSCs were incubated with bFGF and EGF in B27-media. For cell-cycle analysis, cells were treated with a natural enzyme mixture (Accutase; Sigma), washed with 1x phosphate-buffered saline (PBS), fixed, and permeabilized with cold 70% EtOH for 24 hours at -20°C. After incubation, cells were washed three times with 1x PBS and incubated with 500 μl of propidium iodide (BD Biosciences) for 30 minutes at 37°C. Flow cytometric analysis was performed using at least 10,000 cells, and the cell-cycle phases were analyzed using a FACSCalibur flow cytometer (BD Biosciences). For an apoptosis assay, cells were stained with annexin V and 7-AAD (BD Biosciences) in accordance with the manufacturer's instructions. Apoptosis in the cell samples was analyzed at the MD Anderson Flow Cytometry and Cellular Imaging Facility.

### Animal xenografts

In *in vivo* experiments, 4- to 6-week-old female nude mice strictly inbred at MD Anderson and maintained in the MD Anderson Research Animal Support Facility in accordance with Institute of Laboratory Animal Research standards were used. The GSC11 and GSC272 cells (5 × 10^5^) were implanted intracranially into the mice as described previously [45]. The mice were sacrificed at the indicated weeks after tumor-cell implantation, and their brains were removed and processed for analysis. All animal experiments were approved by the MD Anderson Institutional Animal Care and Use Committee. Survival analysis was conducted using the Kaplan-Meier method. Analysis of the mice's tumor volumes was performed using an unpaired two-tailed Student *t*-test, and survival duration of mice was compared using the log-rank test.

### Western blot analysis

GSCs were lysed using RIPA lysis buffer (Cell Signaling Technology) with proteinase (Sigma-Aldrich) and a phosphatase cocktail (Thermo Fisher Scientific). The protein concentration in the supernatant was measured using a BCA protein assay (Bio-Rad). Samples were subjected to sodium dodecyl sulfate-polyacrylamide gel electrophoresis, and the separated proteins were electrophoretically transferred to polyvinylidene fluoride membranes. Blots were incubated with a primary antibody overnight at 4°C and incubated with horseradish peroxidase-linked secondary anti-rabbit or anti-mouse antibodies (Bio-Rad). Antibodies against phosphorylated AKT (cat. #4060), AKT (cat. #9272), phosphorylated p38 (cat. #4511), p38 (cat. #9212), phosphorylated ERK (cat. #9101), ERK (cat. #9102), phosphorylated pyruvate dehydrogenase lipoamide kinase isozyme 1 (PDK1; cat. #3061), PDK1 (cat. #3062), p27 (cat. #13715), phosphorylated FOXO3a (cat. #9465), and FOXO3a (cat. #2497) were purchased from Cell Signaling Technology. CLK2 (cat.#HPA055366) and α-Tubulin (cat. #T9026) were purchased from Sigma-Aldrich. The results of three independent Western blot experiments performed in triplicate were reported.

### Immunohistochemistry and immunofluorescence

Single cells were plated on precoated poly-L-lysine coverslips, fixed in 1% paraformaldehyde for 10 minutes, rinsed with PBS at least three times, blocked in 5% goat serum with 0.2% Triton X-100 for 1 hour, and washed at least three times with PBS and 0.2% Triton X-100. Brain tissue samples from patient were fixed in 4% paraformaldehyde for 24 hours, embedded in paraffin, sectioned serially (4 μm), and stained with hematoxylin and eosin (Sigma-Aldrich). For immunohistochemical staining, slides were deparaffinized and subjected to graded rehydration. After blocking in 3% serum and antigen retrieval (citrate buffer, pH 6.0), the slides were incubated with primary antibodies overnight at 4°C. After the slides were washed in PBS with Tween 20, the primary antibody reactions were detected using a VECTASTAIN ABC Kit (Vector Laboratories) with the respective secondary antibodies. For immunofluorescence studies, after blocking, tissue sections were incubated with an antibody overnight at 4°C. Secondary antibodies (Invitrogen) were incubated for 1hour at room temperature. Antibodies against phosphorylated FOXO3a (cat. #47285; Abcam), CLK2 (cat. #ab188141; Abcam), and Ki-67 (cat. #M7240; Dako) were used in IHC and IF. The results of three independent experiments performed in triplicate were reported.

### Tissue microarray construction and IHC data analysis

The tissue microarray slide was obtained from Dr. Gregory N. Fuller (Department of Pathology, MD Anderson Cancer Center). Paraffin-embedded blocks of 50 human glioblastomas were identified on corresponding hematoxylin- and eosin-stained sections. Normal brain tissue slides were obtained from US Biomax. Immunohistochemical stainings of CLK2 and FOXO3a phosphorylation were scored according to intensity, and glioblastoma tumors were given a score of one to four grades based on staining intensity (0, no staining intensity; 1, weak staining intensity; 2, moderate staining intensity; and 3, strong staining intensity). In the case of heterogeneous sample staining, the higher score was chosen if more than 50% of the cells exhibited greater staining intensity. Survival was calculated as the interval from date of diagnosis (and surgery) to the date of death or last follow-up. Survival analysis was conducted using the Kaplan-Meier method and compared using the log-rank test.

### Detection of multiple signaling pathways using reverse-phase protein array

Vector-infected and CLK2-knockdown GSC272 cells were prepared for reverse-phase protein array (RPPA) analysis. Samples were probed with 279 validated primary antibodies for the analysis at the MD Anderson Functional Proteomics Reverse Phase Protein Array (RPPA) Core facility (http://www.mdanderson.org/education-and-research/resources-for-professionals/scientific-resources/core-facilities-and-services/functional-proteomics-rppa-core/index.html).

### Statistical analysis

All statistical analyses were conducted using the InStat software program for Microsoft Windows (GraphPad Software). Data were reported as the mean ± standard deviation. All other data were compared using an unpaired two-tailed Student *t*-test.

## SUPPLEMENTARY FIGURES



## References

[1] Zheng Y, McFarland BC, Drygin D, Yu H, Bellis SL, Kim H, Bredel M, Benveniste EN (2013). Targeting protein kinase CK2 suppresses prosurvival signaling pathways and growth of glioblastoma. Clin Cancer Res.

[2] Di Maira G, Salvi M, Arrigoni G, Marin O, Sarno S, Brustolon F, Pinna LA, Ruzzene M (2005). Protein kinase CK2 phosphorylates and upregulates Akt/PKB. Cell Death Differ.

[3] Kim H, Choi K, Kang H, Lee SY, Chi SW, Lee MS, Song J, Im D, Choi Y, Cho S (2014). Identification of a novel function of CX-4945 as a splicing regulator. PLoS One.

[4] Sanford JR, Longman D, Caceres JF (2003). Multiple roles of the SR protein family in splicing regulation. Prog Mol Subcell Biol.

[5] Johnson KW, Smith KA (1991). Molecular cloning of a novel human cdc2/CDC28-like protein kinase. J Biol Chem.

[6] Hanks SK, Quinn AM (1991). Protein kinase catalytic domain sequence database: identification of conserved features of primary structure and classification of family members. Methods Enzymol.

[7] Hanes J, von der Kammer H, Klaudiny J, Scheit KH (1994). Characterization by cDNA cloning of two new human protein kinases. Evidence by sequence comparison of a new family of mammalian protein kinases. J Mol Biol.

[8] Araki S, Dairiki R, Nakayama Y, Murai A, Miyashita R, Iwatani M, Nomura T, Nakanishi O (2015). Inhibitors of CLK protein kinases suppress cell growth and induce apoptosis by modulating pre-mRNA splicing. PLoS One.

[9] Rodgers JT, Haas W, Gygi SP, Puigserver P (2010). Cdc2-like kinase 2 is an insulin-regulated suppressor of hepatic gluconeogenesis. Cell Metab.

[10] Nam SY, Seo HH, Park HS, An S, Kim JY, Yang KH, Kim CS, Jeong M, Jin YW (2010). Phosphorylation of CLK2 at serine 34 and threonine 127 by AKT controls cell survival after ionizing radiation. J Biol Chem.

[11] Essers MA, de Vries-Smits LM, Barker N, Polderman PE, Burgering BM, Korswagen HC (2005). Functional interaction between beta-catenin and FOXO in oxidative stress signaling. Science.

[12] Wang MC, Bohmann D, Jasper H (2005). JNK extends life span and limits growth by antagonizing cellular and organism-wide responses to insulin signaling. Cell.

[13] Calnan DR, Brunet A (2008). The FoxO code. Oncogene.

[14] Greer EL, Oskoui PR, Banko MR, Maniar JM, Gygi MP, Gygi SP, Brunet A (2007). The energy sensor AMP-activated protein kinase directly regulates the mammalian FOXO3 transcription factor. J Biol Chem.

[15] Greer EL, Brunet A (2005). FOXO transcription factors at the interface between longevity and tumor suppression. Oncogene.

[16] Brunet A, Bonni A, Zigmond MJ, Lin MZ, Juo P, Hu LS, Anderson MJ, Arden KC, Blenis J, Greenberg ME (1999). Akt promotes cell survival by phosphorylating and inhibiting a Forkhead transcription factor. Cell.

[17] Arden KC (2006). Multiple roles of FOXO transcription factors in mammalian cells point to multiple roles in cancer. Exp Gerontol.

[18] Myatt SS, Lam EW (2007). The emerging roles of forkhead box (Fox) proteins in cancer. Nat Rev Cancer.

[19] Finnberg N, El-Deiry WS (2004). Activating FOXO3a, NF-kappaB and p53 by targeting IKKs: an effective multi-faceted targeting of the tumor-cell phenotype?. Cancer Biol Ther.

[20] Tran H, Brunet A, Griffith EC, Greenberg ME (2003). The many forks in FOXO's road. Sci STKE.

[21] Dijkers PF, Medema RH, Pals C, Banerji L, Thomas NS, Lam EW, Burgering BM, Raaijmakers JA, Lammers JW, Koenderman L, Coffer PJ (2000). Forkhead transcription factor FKHR-L1 modulates cytokine-dependent transcriptional regulation of p27(KIP1). Mol Cell Biol.

[22] Schmidt M, Fernandez de Mattos S, van der Horst A, Klompmaker R, Kops GJ, Lam EW, Burgering BM, Medema RH (2002). Cell cycle inhibition by FoxO forkhead transcription factors involves downregulation of cyclin D. Mol Cell Biol.

[23] Yang JY, Xia W, Hu MC (2006). Ionizing radiation activates expression of FOXO3a, Fas ligand, and Bim, and induces cell apoptosis. Int J Oncol.

[24] Pitts TM, Davis SL, Eckhardt SG, Bradshaw-Pierce EL (2014). Targeting nuclear kinases in cancer: development of cell cycle kinase inhibitors. Pharmacol Ther.

[25] Egozi D, Shapira M, Paor G, Ben-Izhak O, Skorecki K, Hershko DD (2007). Regulation of the cell cycle inhibitor p27 and its ubiquitin ligase Skp2 in differentiation of human embryonic stem cells. FASEB J.

[26] Lee WS, Liu CW, Juan SH, Liang YC, Ho PY, Lee YH (2003). Molecular mechanism of progesterone-induced antiproliferation in rat aortic smooth muscle cells. Endocrinology.

[27] Yu CH, Wu J, Su YF, Ho PY, Liang YC, Sheu MT, Lee WS (2004). Anti-proliferation effect of 3-amino-2-imino-3,4-dihydro-2H-1,3-benzothiazin-4-one (BJ-601) on human vascular endothelial cells: G0/G1 p21-associated cell cycle arrest. Biochem Pharmacol.

[28] Rodgers JT, Vogel RO, Puigserver P (2011). Clk2 and B56beta mediate insulin-regulated assembly of the PP2A phosphatase holoenzyme complex on Akt. Mol Cell.

[29] Colwill K, Pawson T, Andrews B, Prasad J, Manley JL, Bell JC, Duncan PI (1996). The Clk/Sty protein kinase phosphorylates SR splicing factors and regulates their intranuclear distribution. EMBO J.

[30] Duncan PI, Stojdl DF, Marius RM, Scheit KH, Bell JC (1998). The Clk2 and Clk3 dual-specificity protein kinases regulate the intranuclear distribution of SR proteins and influence pre-mRNA splicing. Exp Cell Res.

[31] Vousden KH, Lu X (2002). Live or let die: the cell's response to p53. Nat Rev Cancer.

[32] Beaulieu JM, Sotnikova TD, Marion S, Lefkowitz RJ, Gainetdinov RR, Caron MG (2005). An Akt/beta-arrestin 2/PP2A signaling complex mediates dopaminergic neurotransmission and behavior. Cell.

[33] Cancer Genome Atlas Research N (2008). Comprehensive genomic characterization defines human glioblastoma genes and core pathways. Nature.

[34] Nakamura N, Ramaswamy S, Vazquez F, Signoretti S, Loda M, Sellers WR (2000). Forkhead transcription factors are critical effectors of cell death and cell cycle arrest downstream of PTEN. Mol Cell Biol.

[35] Sunayama J, Sato A, Matsuda K, Tachibana K, Watanabe E, Seino S, Suzuki K, Narita Y, Shibui S, Sakurada K, Kayama T, Tomiyama A, Kitanaka C (2011). FoxO3a functions as a key integrator of cellular signals that control glioblastoma stem-like cell differentiation and tumorigenicity. Stem cells.

[36] Naka K, Hoshii T, Muraguchi T, Tadokoro Y, Ooshio T, Kondo Y, Nakao S, Motoyama N, Hirao A (2010). TGF-beta-FOXO signalling maintains leukaemia-initiating cells in chronic myeloid leukaemia. Nature.

[37] Hu MC, Lee DF, Xia W, Golfman LS, Ou-Yang F, Yang JY, Zou Y, Bao S, Hanada N, Saso H, Kobayashi R, Hung MC (2004). IkappaB kinase promotes tumorigenesis through inhibition of forkhead FOXO3a. Cell.

[38] Modur V, Nagarajan R, Evers BM, Milbrandt J (2002). FOXO proteins regulate tumor necrosis factor-related apoptosis inducing ligand expression. Implications for PTEN mutation in prostate cancer. J Biol Chem.

[39] Shi J, Zhang L, Shen A, Zhang J, Wang Y, Zhao Y, Zou L, Ke Q, He F, Wang P, Cheng C, Shi G (2010). Clinical and biological significance of forkhead class box O 3a expression in glioma: mediation of glioma malignancy by transcriptional regulation of p27kip1. J Neurooncol.

[40] Sherr CJ (1996). Cancer cell cycles. Science.

[41] Kossatz U, Malek NP (2007). p27: tumor suppressor and oncogene …?. Cell research.

[42] Xiong Y, Hannon GJ, Zhang H, Casso D, Kobayashi R, Beach D (1993). p21 is a universal inhibitor of cyclin kinases. Nature.

[43] Lee J, Kim SS (2009). The function of p27 KIP1 during tumor development. Exp Mol Med.

[44] Vervoorts J, Luscher B (2008). Post-translational regulation of the tumor suppressor p27(KIP1). Cell Mol Life Sci.

[45] Piao Y, Liang J, Holmes L, Henry V, Sulman E, de Groot JF (2013). Acquired resistance to anti-VEGF therapy in glioblastoma is associated with a mesenchymal transition. Clin Cancer Res.

