# Supplementary material for: Cdc2-like kinase 2 is a key regulator of the cell cycle via FOXO3a/p27 in glioblastoma
Source: Oncotarget. 2016 Mar 30;7(18):26793–805. doi: 10.18632/oncotarget.8471 (PMC5042015; doi:10.18632/oncotarget.8471)
Supplement: Supplementary file 1 [file oncotarget-07-26793-s001.pdf]

SUPPLEMENTARY FIGURES

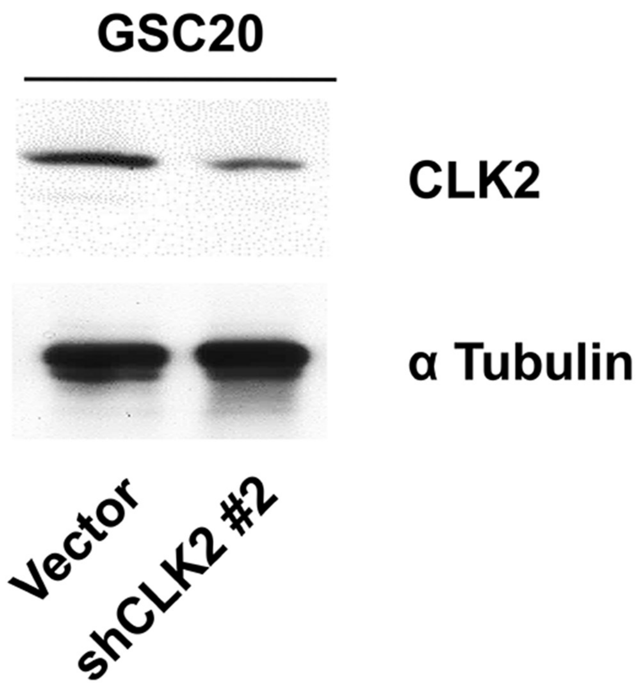

Supplementary Figure S1: Knockdown of CLK2 expression in GSC. GSC20 was infected with a lentivirus containing an empty vector or CLK2 shRNA2. Cell lysates were collected, and the CLK2 expression was measured using Western blot.

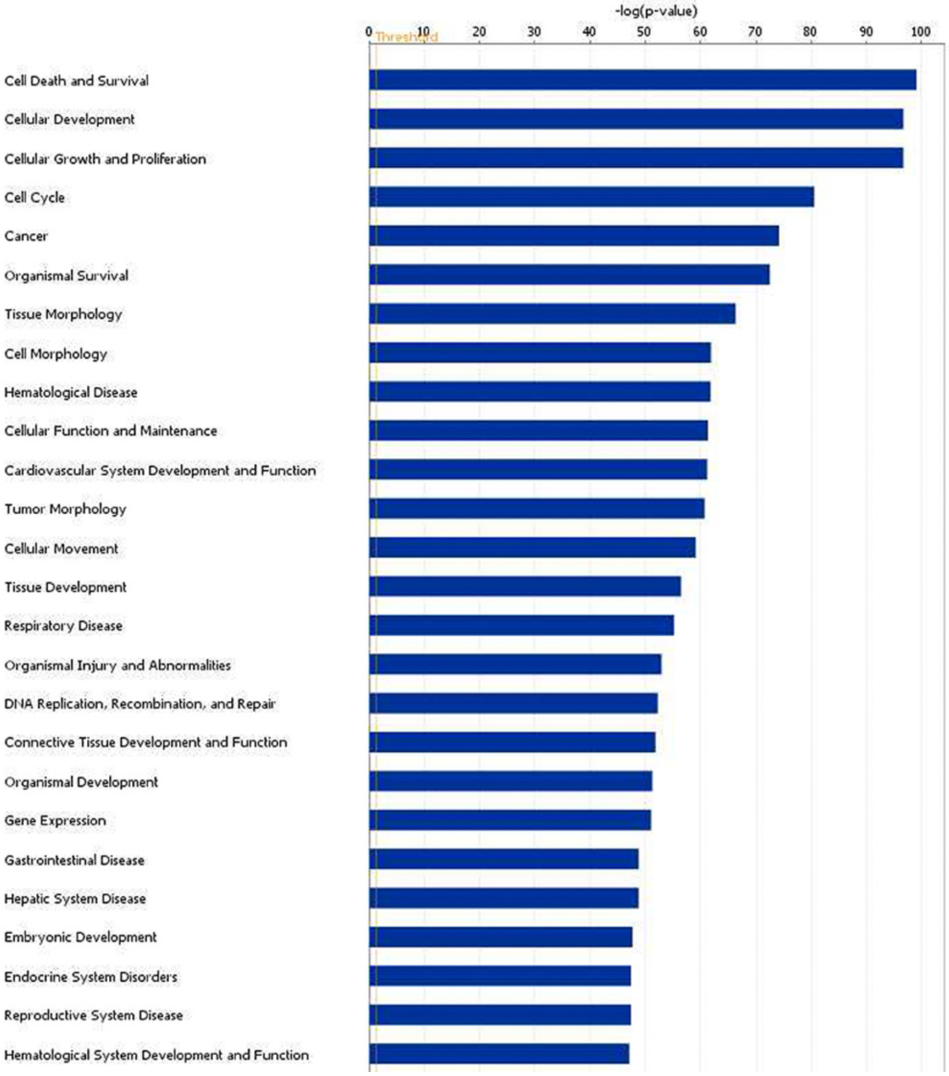

Supplementary Figure S2: Biological function of CLK2 in GSC272 cells. RPPA analysis was performed and the biological functions enriched in genes differentially expressed in CLK2-knockdown compare to vector-infected GSC272 cells was assessed using Ingenuity Pathway Analysis.

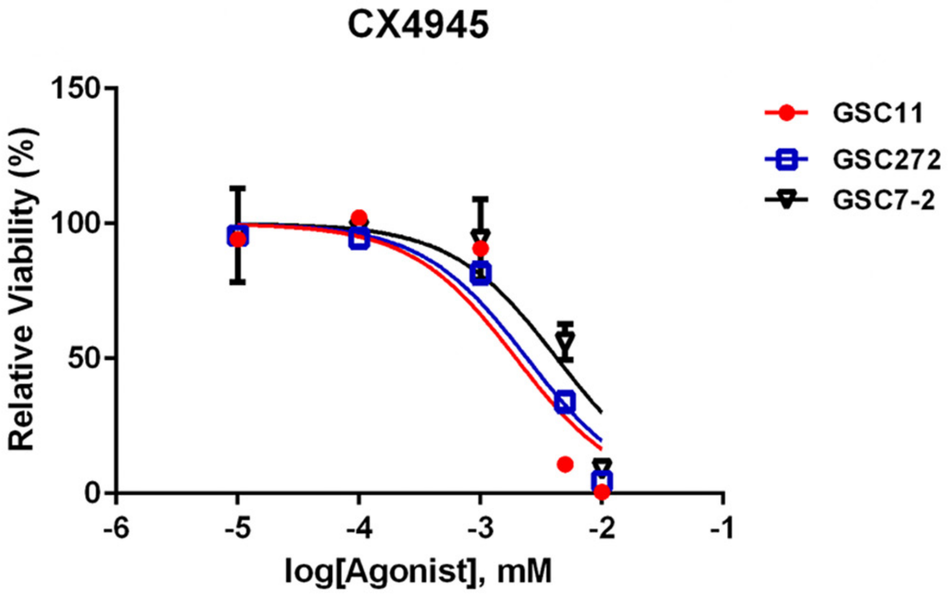

Supplementary Figure S3: CX4945 decreased the viability of GSCs. The cells were treated with CX-4945 as selective inhibitor of CK2a for 5 days. CellTiter-Glo Reagent was added to cells, and the luminescence was recorded.

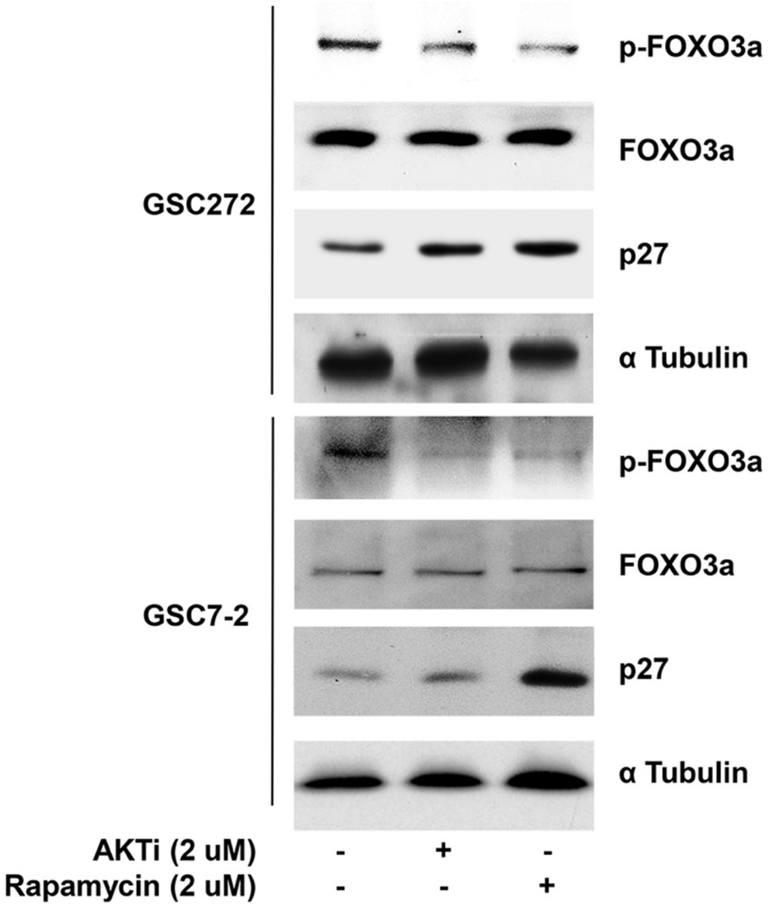

Supplementary Figure S4: Decreased phosphorylation of FOXO3a and p27 resulting from inhibition of the AKT and mTOR pathways. GSC272 and GSC7-2 cells were pretreated with inhibitors for 2 hours and incubated with bFGF and EGF for 24 hours. Expression of phosphorylated FOXO3a and p27 protein was detected using Western blot.
